# Perspectives on Psychometrics Interviews with 20 Past Psychometric Society Presidents

**DOI:** 10.1007/s11336-021-09752-7

**Published:** 2021-03-26

**Authors:** Lisa D. Wijsen, Denny Borsboom

**Affiliations:** Department of Psychological Methods, University of Amsterdam, Nieuwe Achtergracht 129-B, 1018 WS Amsterdam, The Netherlands

**Keywords:** history of psychometrics, oral history, interviews, qualitative research, statistics

## Abstract

In this article, we present the findings of an oral history project on the past, present, and future of psychometrics, as obtained through structured interviews with twenty past Psychometric Society presidents. Perspectives on how psychometrics should be practiced vary strongly. Some presidents are psychology-oriented, whereas others have a more mathematical or statistical approach. The originally strong relationship between psychometrics and psychology has weakened, and contemporary psychometrics has become a diverse and multifaceted discipline. The presidents are confident psychometrics will continue to be relevant but believe psychometrics needs to become better at selling its strong points to relevant research areas. We recommend for psychometrics to cherish its plurality and make its goals and priorities explicit.

**Supplementary Information:**

The online version supplementary material available at 10.1007/s11336-021-09752-7.

In the history and sociology of science, the focus often lies on a chronology of important events, such as the making of significant discoveries and the emergence of scientific theories. When there is mention of the persona behind these discoveries, studies often stress the scientist’s contributions. In the history of science, the emphasis thus often lies on specific contributions and written sources, such as research articles and dissertations. Examples of such studies on the history of psychometrics are Bennett and Von Davier (2017), Jones and Thissen (2007), Van der Heijden and Sijtsma (1996), and Wijsen et al., (2019). However, scientists are not only tied to their discoveries or theories; they often entertain thoughts and visions about how science should operate, which cannot always be found in these written sources. Due to their close involvement in a specific research area, it is likely that researchers have relevant ideas about historical, current, and future developments. This article presents exactly those thoughts and visions of researchers, psychometricians in our case, about the historical, current, and future directions of their field. This article thus sheds light on the first-person narratives that most historical studies usually overlook.

This project was inspired by the research methodology of oral history. Oral history is a branch within historical research that focuses on collecting personal testimonies of people who have witnessed a particular period or event (Abrams, 2010; Thompson, 2017). Oral history studies move the focus from written archival sources to the memories of people and invite these people to share their memories in interviews. In the history of science specifically, oral history invites scientists to share their memories of doing research and shed light on dilemmas and choices they encounter in their daily jobs. Such projects are becoming increasingly popular. Particularly in the USA, many university libraries and research institutes now have access to large collections of interviews with scientists, predominantly from the physical sciences (Doel, 2003). Other examples of oral history projects in science are Wright and Ville (2017) in economic history, Baer et al. (1991) in political science, and Smith and Rennie (2014) in evidence-based medicine.

With this oral history project, our aim was to provide a detailed and nuanced account of the history of psychometrics by asking prominent psychometricians to share their knowledge and memories of their personal career and the history of psychometrics. However, an oral history project also presents the opportunity to look at history that is actually in the making (Weiner, 1988). Most of our interviewees are still active in psychometric research or practice, and those that are not are often still involved or at least interested in current developments in psychometrics. So, not only do the interviews gain access to knowledge of psychometrics’ history that otherwise might have gotten lost, the interviews enabled us to find out how psychometricians perceive current and future developments in psychometrics. Our oral history project thus investigates both the history of the field and psychometricians’ perspectives on current and future directions.

Groenen and Van der Ark (2006) describe the interviews they held with 12 prominent psychometricians with the purpose of investigating the current status of psychometrics. These interviews focused specifically on specific models and techniques that have either been influential historically speaking (such as Item Response Theory or Structural Equation Modeling) or interesting developments in contemporary or future psychometrics, such as data mining and Bayesian analysis. Though several of these developments are also mentioned by our interviewees in the interviews on several occasions, the focus of our analysis lies less on describing these concrete examples of models and research traditions, and more on the underlying motivations and reasons *why* psychometricians do research in a particular way. So, besides having a descriptive purpose, our paper also aims to analyze the given answers on a deeper level. For example, can we distinguish different types of approaches of doing psychometrics, and how do these approaches contradict each other? What are the different attitudes we find in relation to the future of the field, or with respect to other research areas? The qualitative analysis in this paper thus aims to uncover the different perspectives on psychometrics held by our interviewees.

One of the reasons it is particularly relevant to ask psychometricians to reflect on their own research domain is because psychometrics has a complicated position with regard to its close neighbors: psychology and statistics (Borsboom, 2006; Groenen & Van der Ark, 2006; Sijtsma, 2006). Psychometrics’ origins may be nested in psychology, but its current course diverges in many directions (one of them being statistics), and this results in a multitude of approaches and perspectives on what psychometrics should offer. Should psychometricians affiliate more with the psychologists and work on building psychological theory and explaining human behavior, or should they focus on designing statistical methods that are valuable to export to other fields as well? And what do they believe will happen to psychometrics in the following decades? Do psychometricians expect psychometrics to remain a successful research area in the future, or are there challenges ahead which psychometrics first needs to overcome?

In this project, we invited psychometricians to share their perspectives on such questions regarding the past, present, and future of psychometrics. The interviews provided a wealth of historical knowledge and interesting ideas that we cannot all incorporate in this article. For the sake of openness of data and preserving the richness of the interviews, we decided to compile the revised transcripts in a book (OMITTED FOR REVIEW, forthcoming) so that the entire interviews will be accessible to people who are interested in reading the stories of our presidents. This article though is a more in-depth qualitative analysis of these interviews and addresses several of the topics, themes, and dilemmas that are important to the presidents and the authors. Ultimately, we show how diversely psychometricians perceive their own field, and that psychometrics is not restricted to one approach only. In the discussion, we elaborate on how the interviews inspire a range of historical and philosophical questions for further research.

## Methods

We invited 36 presidents of the Psychometric Society to participate as respondents in our project. The rationale for this choice lies in the fact that presidents of the Psychometric Society are key figures in psychometric research and are democratically chosen by the psychometric community; their reflections on psychometrics are therefore intrinsically interesting and worth preserving. We approached the presidents through a personal invitation, in which we asked the presidents to contribute to an oral history project about the history of psychometrics. Twenty-one presidents accepted our invitation; one president eventually canceled the appointment. A small possible source of selection bias is that our location, the Netherlands, and our attendance at the IMPS meeting in Asheville, North Carolina, made it relatively easy to interview people who reside in the Netherlands or who attended this conference. We also see that the older presidents were more inclined not to accept or respond to our invitation. Reasons for declining our interview were geographic location, old age, or not considering it an important cause. Some presidents did not respond to our invitation. The 20 interviewees who accepted our invitation were president of the Psychometric Society for a period of 1 year sometime between 1982 and 2013.

All interviews were held in person, either at people’s homes, their work offices, in a public space, or at the International Meeting of the Psychometric Society (IMPS) in 2016 in Asheville, North Carolina. The interviews took place between April 2016 and October 2017. The interviews took between 45 min and one hour and were videotaped. The interviewee signed an informed consent in which he or she consented to use the material for this research project.

The questions of the interview were built around four topics: the respondents’ professional career, their views on the relationship between psychometrics and other scientific disciplines, the history of psychometrics, and future directions of psychometrics. The questions were organized in a semi-structured interview format, which served as a general guideline. A subset of questions was posed to all candidates, but each interview allowed enough space and time to discuss topics that were interviewee-specific. The questions for the interview were sent to the interviewee beforehand if so requested.

The interviews were first transcribed in Inqscribe (Inquirium, 2013) and then roughly edited: The edited texts are as close as possible to the original transcriptions^1^ but modified into readable and accurate English. When we were not completely certain about the exact wording used by the interviewee, we contacted the president and asked for rectification. The quotes from the interviews in this article are selected from these modified versions. For the sake of accuracy, the quotes were not taken from the more thoroughly revised versions that will be included in the compilation. After editing, we performed a qualitative analysis of the interviews: We identified the most prevalent themes, partly based on the themes already provided by the questions, partly based on the input by the presidents, and collected sections from the transcriptions for each individual theme. A selection of themes and corresponding quotes we thought were most relevant is discussed in the results below.

## Themes

Improving our understanding of the history of psychometrics was the main reason for doing an oral history project. Before we continue with the presidents’ perceptions, we will sketch a general (historical) framework that helps to contextualize the interviews.

Psychometrics originated at the end of the nineteenth century and early twentieth century, with the work of academics like Francis Galton, Karl Pearson, Charles Spearman, and Louis L. Thurstone. It has seen a number of shifts which closely resemble the four generations of test theory that Paul Holland (one of our interviewees) has conceptualized (Dorans, 2011). Holland’s delineation starts in the early twentieth century when test theory’s first generation started with developments in classical test theory, reliability, and validity. The second generation, which started in the 1940s and peaked in the 1970s, was concerned with the development of models for item-level data. The third generation, which started in the 1970s, focused on the statistical advancement of item-level models. The fourth generation attempts to bridge the gap between the psychometrician and the testing enterprise, by developing methods for differential item functioning or test equating.

When we transpose this delineation to psychometrics, we find that it lacks a clear role for factor analysis (a first-generation development and, as we will see later, considered crucial in the history of psychometrics) and structural equation modeling and multidimensional scaling as part of the second and third generation. Moreover, we consider the fourth generation to be broader than just bridging the gap between psychometrics and the testing enterprise: As we will see below, many fourth-generation psychometricians aim at finding connections with a variety of other sciences and enterprises, not just the testing industry.

Importantly, Holland argues that none of these generations have permanently ended: All generations—though some might have drastically shrunk over the years—are still active research domains, and *Psychometrika* still publishes research from these four domains. The most cited papers from the past two decades concern topics in structural equation modeling, reliability estimates, and advances on a variety of latent variable models (a mixture of topics from different generations). Articles on ”Item Response”. theory still make up a significant part of *Psychometrika’s *content, and historically speaking, articles on the analysis of proximities have also been one of *Psychometrika’s* pillars (Heiser et al., 2016). More recent directions are cognitive diagnosis, Bayesian methods for model estimation, and computer adaptive testing. What is interesting about this list is that topics like the replication crisis, questionable research practices, and the practice of educational measurement—exceptions granted—are usually not addressed in *Psychometrika*. *Psychometrika* mainly publishes in-depth theoretical and technical papers, not commentaries on research or testing practices. Psychometrics, as understood in this paper, is thus a highly technical, abstract, and model-based research domain.

### Key Moments in the History of Psychometrics

In the interviews, we asked the presidents how they perceive the history of psychometrics, and especially what they believe were psychometrics’ key moments and main achievements.

One of the questions we asked was what the presidents believe is the most significant work or the most important psychometrician in the history of psychometrics. The most common answer (given by eight interviewees) was that this must be Lord & Novick’s *Statistical Theories of Mental Test Scores* (1968). *Statistical Theories of Mental Test Scores* came out at ETS (Bennett & Von Davier, 2017) and was one of the first works in psychometrics to give a formal treatment of classical test theory (Traub, 1997). Its publication took place in the midst of the shift from classical test theory to modern test theory, possibly the quintessential paradigm shift in psychometrics. Though classical test theory was strictly speaking never falsified, the latter became dominant in most psychometric research. Lord & Novick (1968) is one of the first comprehensive works to treat topics from both classical and modern test theories. Brian Junker praises it for having ‘everything from factor analysis to IRT and other things that are relevant to standard measurement questions in psychometrics. [...] there is a real effort to connect psychometrics to current thinking in statistics.’ Ivo Molenaar praises it for being:

on the transition of the old classical correlation-based and classical test theory-based models, to the item response models and latent trait models. [...] Fred Lord was the classical one, and Mel Novick brought in the logistic models, which was definitely a very important step for the psychometric community as a whole.

This strong consensus on the central importance of Lord & Novick’s *Statistical Theories of Mental Test Scores *is remarkable and invites further research on the effect the work has had on the development of the field.

Some presidents go further back in time to the early twentieth century and consider either Charles Spearman or Louis L. Thurstone, the founders of factor analysis, as the most important psychometrician in the history of psychometrics. Klaas Sijtsma regards Spearman as revolutionary:

He actually combined psychological problems he was struggling with, with the development of statistical tools that he needed to tackle those problems, and in a way, he is the founding father of classical test theory and factor analysis, which is not a small accomplishment; it is incredible.

Paul De Boeck states that, between Charles Spearman and Louis Thurstone, he prefers the latter. Thurstone (1934) ‘He [Louis Thurstone] was doing factor analysis, but not just to measure. His paper was called ‘Vectors of Mind’, so he wanted to explain the human mind. He both had an interest in measurement, and an interest in understanding how the mind functions.’ Larry Hubert commends Thurstone for training and educating so many prominent psychometricians, like Paul Horst and Ledyard Tucker. And it was also Thurstone whom David Thissen admires most:

Thurstone made everything. Thurstone made the discipline; he came from nowhere, received degrees in things like engineering, and created quantitative psychology; he created scaling, he changed factor analysis into multiple factor analysis. He started the Psychometric Society.

Willem Heiser and Robert Mislevy consider Lee Cronbach as one of the most influential psychometricians in history. According to Heiser, Cronbach’s paper on the reliability coefficient is one of his most significant contributions (Cronbach, 1951), due to its applicability to practical problems in research, not only in psychology but also in medical science or other fields where measurement plays a central role. Mislevy praises Cronbach for thinking critically about psychological measurement and the inferences or conclusions you can draw based on certain data, referring here to generalizability theory (Cronbach et al., 1972): ‘he laid down some real mileposts, about how psychometrics is not just about measurement, it is about the quality and the nature of inferences that you’re making.’

Some presidents do not mention specific people, but rather focus on a typical psychometric idea that was historically significant. For example, Peter Bentler mentions the theory of error as an essential scientific contribution by psychometrics:

Very influential was the idea of errors in measurement, which of course, had been around for a long time in astronomy – it is not like Spearman invented it - but Spearman thought about it in a way that made it relevant to psychological measurement.

Jos ten Berge agrees: ‘The very simple fact that when you measure someone’s intelligence twice, you don’t get the same results, means that at least one of the two measurements cannot be correct, and that must be error.’ Not only is the idea of the quantification of error in measurement an important scientific contribution of psychometrics, but it also marks the attitude of the psychologist or psychometrician as a researcher. Jos ten Berge argues the following:

It is a very interesting fact that psychologists have a routine of evaluating their measurements, for instance, by reliability and validity studies. It is a form of self-criticism that often isn’t sufficiently appreciated. It is a very beautiful situation: a discipline that distrusts its own results.

The conceptualization of measurement error and its incorporation in psychometric models are thus seen as unique contributions of psychometrics to the sciences. Moreover, these contributions characterize how the psychometrician practices research: with a strong awareness of the imperfection of (psychological) measurement. Ten Berge’s remark underscores that the characteristic viewpoint of the psychometrician involves the recognition and appreciation of the problems involving psychological and educational measurement.

### The Dark Ages of Psychometrics

According to several presidents, psychometrics’ most important contribution to society is psychological and educational testing. Testing has pervaded several phases in people’s lives, and psychometricians turned it into a standardized and reliable enterprise. However, measurement and testing do not only resonate in the ears of some of our respondents as something that is only positive and for a good reason. Despite the fact that the controversial part of the history of psychometrics was not an official interview topic, some presidents bring it up themselves, often torn between psychometrics’ controversial history on the one hand and its important achievements on the other. When David Thissen states that it was indeed testing that put psychometrics on the map, twice, he states that this was ‘for better or for worse.’ Jacqueline Meulman says that she:

was amazed by how many bad things had happened in psychometrics, I was flabbergasted. On the other hand, I was intrigued by the mathematical background of the methods I was reading about [...]. Although I did realize that many of the great psychometricians didn’t have very good political backgrounds, I was intrigued by the methods themselves [...].

The interviewees refer here to the controversial history of mental measurement, which was strongly intertwined with nineteenth and twentieth-century politics, and especially eugenics. Eugenics—a scientific and political movement that aimed to improve the genetic quality of the human population, which thrived late 19th and early twentieth century (Chitty, 2007)—was a popular ideology among many psychometricians, among which Charles Spearman, Lewis Terman, and James McKeen Cattell. In these times, the measurement of intelligence was often misinterpreted and misused to attribute differences in intelligence test scores to genetics (Jackson & Weidman, 2004; Richards, 2012). Predominantly during the late nineteenth century and early twentieth century (though not exclusively so), differences in scores on intelligence tests served as ‘scientific’ proof for the claim that some groups (Afro-Americans, women, people of lower classes) were less intelligent and thus less worthy than upper-class white males. And though the Psychometric Society did not have an explicit eugenic ideology (or any political motivation for that matter), at least one president entertained similar ideas. Henry Garrett, president in 1943, supported the idea of hereditary racial differences in intelligence and racial segregation (Winston, 1998). The history of psychometrics is thus not a sequence of one groundbreaking scientific achievement after the other, nor were all psychometricians always distrusting of their results.

Other presidents also refer to the adverse effects of psychometric research. Bill Stout states that when done well, psychometrics can be very important, but psychometricians have also sometimes ‘oversimplified a very complicated subject.’ Here, Stout refers to the Bell Curve controversy (Herrnstein & Murray, 1994), a more recent example of how differences in intelligence scores are used to justify differences between races and social groups. Larry Hubert is highly critical of psychometrics’ past, and where other presidents see testing as a relatively positive contribution of psychometrics, Hubert is not so sure: ‘[...] I’m not sure if all in all the idea of measuring intelligence hasn’t brought more ill stuff than it has brought good stuff. The whole politics of race and psychometrics is not a very happy one.’ Though the dark ages of psychometrics were not an official interview topic, several presidents touch upon them on their own initiative, implying that these dark ages should not be overlooked in further historical research.

### The Relationship Between Psychometrics, Psychology, and Statistics

As we discussed in the introduction, what is intriguing about psychometrics is its position relative to other disciplines. Though psychometrics originated in psychology, it is now closely affiliated to statistics as well. In this section, we will discuss how the presidents perceive the relationship between psychometrics and two of its closest neighbors: psychology and statistics.

#### Psychometrics, Psychology, and Educational Measurement

The relationship between psychometrics and psychology is hard to define, but the detachment between psychometrics and psychology (and also the detachment between psychometrics and educational measurement) rises to the surface in several interviews. What the psychometricians disagree on is whether this detachment is indeed an issue, and in case it is how psychometricians should act on it.

A particularly vivid illustration of the disconnected relationship between psychology and psychometrics is formed by the similarly detached attitude of some of the interviewees towards psychology. Some presidents express a certain ignorance of or lack of interest in what is going on in psychological research: They explicitly mention knowing little of psychology, or just not being interested in it. For example, statistician Bill Stout stresses the importance of statistics in psychological research but mentions not knowing enough what is going on in the field of psychology to see how psychometrics can contribute. Jacqueline Meulman expresses her discomfort with topics in psychology or educational measurement and states she feels more at home in biostatistics. Though appreciative of fellow psychometricians doing psychological research, their own interests lie somewhere else.

This indicates an important change with respect to the early twentieth century because it is hard to imagine a similar approach to psychology and psychometrics in the early days of psychometrics when psychometrics and psychology were still in a close relationship. The remarks of some of the presidents show that it is currently possible to be a successful psychometrician and a president of The Psychometric Society, without having either a background or an active interest in psychology. Being successful in psychometrics and being a president of the Psychometric Society, therefore, does not require a strong connection to psychology or educational measurement: Having strong ties with mathematics or biostatistics is equally relevant and appropriate. Modern psychometrics has thus evolved into a field that is no longer dedicated to psychology alone and can no longer be defined as psychology’s statistical counterpart; instead, psychometrics has developed ties with different fields, which shows in the backgrounds and interests of the presidents of the Psychometric Society.

Several presidents argue that standardized testing or educational measurement is the most important contribution of psychometrics. However, some stress that psychometrics also has trouble reaching educational measurement: Similar to psychology, educational measurement is missing out on some of the newest psychometric methods. Susan Embretson explains that this is because ‘testing is the hardest thing to change’; people in education are slow in adopting cognitive theory for item construction. According to Jacqueline Meulman, educational measurement is missing out on psychometrics because ‘major testing institutes in the US don’t use the work of psychometricians, and there are even institutes or agencies that do testing that use nothing that comes of out of the psychometric community.’ However, the detachment might be less severe than with psychology: psychometricians like Wim van der Linden and Hua-Hua Chang also see many possibilities for psychometrics in educational measurement, especially for adaptive testing. According to Van der Linden and Chang, there is high demand for adaptive methods and they see this continuing in the future.

There are a number of possible explanations for the growing distance between psychology and psychometrics. David Thissen explains that, before the 1950s, a psychologist was also trained in psychometrics, but for the sake of the grant system, psychology departments are divided into subfields. ‘It is now almost inconceivable to get to this state of the art in more than on one of these subareas, in one brain. You can never know enough.’ In other words, one becomes a social psychologist, a developmental psychologist, or a psychometrician, and there is very little mingling between the three professions. Related to this, Jan de Leeuw states that he also finds it the job of the psychologist, not of the psychometrician, to engage with building psychological theories. According to De Leeuw, the psychologist and the psychometrician simply have different job descriptions, which means that the work they are doing is fundamentally different.

A second explanation has to do with how psychometric research is communicated to external parties. Bengt Muthén, Larry Hubert, and Peter Bentler express their opinion that *Psychometrika* or other psychometric literature can sometimes be too narrow in terms of content, and perhaps also too technical and too theoretical for the psychologist or educational researcher to read and use. Consequently, *Psychometrika* has become out of reach for applied researchers without thorough psychometric or statistical training. Psychometrics might thus have become too much of a niche, and consequently, detached from psychology.

#### Psychology first!

For several presidents, the growing distance between psychology and psychometrics is a reason to worry. Klaas Sijtsma states that he now encourages ‘everybody to engage in theory building. So, to become a psychologist, rather than a psychometrician.’ He pleads for a more unified psychology, where once again people are trained both as a psychometrician and psychologist. De Boeck also pleads against using psychometrics as purely a statistical toolkit. ‘I think psychometrics is a way of thinking about substantive issues, and it’s possible to come up with ideas, substantive ideas, based on a certain way of understanding psychometric models.’ According to these presidents, psychometrics is not just a toolbox of purely statistical, data-analytic models, but a set of models and techniques that can inspire substantive thinking about psychological problems and thereby aid psychology theory building.

A reason why building psychological theory is no longer one of psychometrics’ priorities is given by Susan Embretson:

There is a whole breed of psychometricians out there who seem to have less of a substantive background, and I do not think that’s a good thing. I think they might be dealing with rather narrow statistical issues that are not really going to make a difference in the discipline [...]. So, I really see a necessity to keep quantitative methods attached to a discipline so it can influence that discipline.

According to Embretson, psychometricians can sometimes be too involved with technical details, whereas they should pay more attention to what they can contribute to psychological research. As mentioned earlier, *Psychometrika* mostly publishes articles on narrow, statistical issues, rather than articles that are relevant and readable for the psychologist. Psychologists might, therefore, not be inclined to look for relevant literature there.

However, the reason for the detachment does not only lie in psychometrics’ court. Several presidents mention the lack of interest of the psychologist in applying proper psychometrics. When we ask James Ramsay to identify the relationship between psychology and psychometricians, he answers:

I would say it is both distant and uneasy because the psychologist needs psychometricians badly, but quite frankly, once they have what they need, they do not want to hear anything else, so statistically speaking, it is a very conservative community.

It is hard to escape a sense of disappointment or frustration here. Psychometricians are not able to get their expertise across, whereas helping psychologists with their methodological problems is often considered part of the job description of the psychometrician. The psychometrician is supposedly the consultant who offers statistical or methodological advice, but psychometricians can only do their job if the psychologist seeks the psychometrician’s help when in need. In practice, this does not happen frequently enough, and that is a shame. Wim van der Linden states that psychometricians ‘could be a major support to psychology, make their measurement rigorous, and then plan their experiments better, help them model. [...] it could feed psychology.’ Psychometrics could thus provide valuable input for the psychologist, which the psychologist is now missing out on.

The interviews show that the relationship between psychology and psychometrics is nothing short of complicated. What makes the psychology–psychometrics relationship even more challenging is that psychometrics is also strongly affiliated with statistics, the topic of the next section.

#### Psychometrics and Statistics

After psychology, statistics is probably psychometrics’ closest kinship, and the relationship between the two was frequently touched upon in the interviews. According to Brian Junker, the separation of psychometrics and psychology is not necessarily a reason to worry: ‘In a certain sense, psychometrics is by definition tied to psychology, but the methods are really just the methods of latent variable modeling for individual differences, and that may or may not be tied to psychology.’ According to Junker, psychometrics may have its origins in psychology, but this does not imply that psychology should be its only connection. Many presidents stress that it would be beneficial for psychometrics if it were to extend its influence to other fields. They believe psychometrics should make more effort to be taken seriously by other fields, like statistics, since it could make important contributions there as well.

Willem Heiser uses the metaphor of a river system to describe the relationship between statistics and other disciplines with a strong quantitative component:

A river system starts with small little rivers, and which is where I consider the various disciplines, like biology, psychology, economy, econometrics, chemistry. Those are the areas where people do quantitative things. Sometimes, they invent something for themselves which is useful for others, and then these techniques that are invented in a substantive area go down the stream to the big river. The big river is statistics, so to speak. That is where everything ends up.

According to Heiser, scientific disciplines with a quantitative focus each develop their own statistical methods, which at first are devoted to solving a specific substantive research question, but then get stripped from substantive interpretation. These models are subsequently free to move from the small river to the big river of statistics, which is filled with models developed in a wide variety of research areas. Not uncommonly, quantitative methods developed in one river find their way to other disciplines as well. An example of such a method in psychometrics would be factor analysis, which was originally developed to describe general intelligence, and has now found its way to other research areas both in and outside psychology (Young & Pearce, 2013).

The close connection between statistics and psychometrics becomes clear when we find that a number of presidents do not have a background in psychology, but in statistics or mathematics. Paul Holland articulates this close connection between the two: ‘I think that psychometrics has a very strong statistical side, I keep thinking of psychometrics as being part of statistics, not so much “psycho”. Even though the guys that invented the field all came from psychology.’ Like Willem Heiser, Paul Holland stresses that methods developed in psychometrics are no longer restricted to psychological research alone and can be used by other disciplines. Taking Holland’s perspective a bit further, we might say that psychometrics has lost its ‘psycho’-affiliation throughout the years and became a type of modeling that is relevant for a variety of research domains (psychology, sociology, medical science, artificial intelligence) and can be gathered under the statistics umbrella.

Even though psychometrics and statistics have a close relationship, several presidents point out that psychometrics has a problem making that connection beneficial for both sides: There is plenty of proper, technically well thought out psychometric work that is useful for the statistician but is not recognized as such by other statisticians. Jan de Leeuw gives a reason why original psychometrics did not strike a chord with the statisticians: ‘It was mostly because of the way the original factor analysts, who were psychologists, like Spearman and Cattell, presented [factor analysis] as some magical tool that could discover laws of nature by simple inductive data analysis.’ Interestingly, the same magic-jargon is mentioned by Bengt Muthén, who says that ‘statisticians think of that [factor analysis and structural equation modeling] as hocus pocus machinations.’ Psychometricians magically pulling ‘factors,’ such as intelligence, out of the hat did not sit well with the statisticians, who were possibly less interested in making strong substantive claims about the identity of latent variables than the psychometricians and psychologists at the time.

Moreover, some interviewees point out that on a number of occasions, research that was being done under the name of statistics, had actually already been done before in psychometrics. But because psychometrics is too much of a niche field, researchers from other fields simply do not know it had already been done before. And this leads to frustration among some of the presidents since psychometrics could, in fact, contribute a lot to the field of statistics. According to Muthén:

[...] it is a strong tendency in statistical journals to refer to early statistical articles referring to the psychometric literature [instead of referring directly to the original psychometric literature] [...]. It seems psychometric publishing seems to be too separatedfrom general mainstream statistical modeling [...].

Interestingly, the public relations issues of psychometrics seem to come up both with the psychology-oriented presidents and with the statistics-oriented presidents: Psychometricians are not able to reach out to either group and fail to receive acknowledgment for their work.

### The Identity of the Psychometrician: A Multitude of Approaches

The sections above show there are multiple ways how the psychometricians perceive their own field, and that contemporary psychometrics consists of a variety of approaches, each with their own ideas and visions. Below, we distinguish between five approaches we have recognized in the interviews. Our intention here is not to categorize each respondent and define them as a specific type of researcher, but to show there are different ways in which psychometrics research can or should be practiced, each prioritizing different characteristics or elements of psychometric research. The types discussed below underscore the plurality of approaches in a field that, to the outside, might seem relatively uniform.

#### The Psychologist

First of all, unsurprisingly perhaps, we identify the psychometricians who identify themselves as both a psychometrician and a psychologist. The psychology-oriented psychometrician uses psychometrics as a way to improve psychological understanding and always has a substantive interest. According to the psychology-oriented perspective, psychometric models do not only describe or summarize psychological data but can help in understanding or explaining the data as well. The division between the psychometrician and the psychologist then becomes rather fuzzy: Psychometricians who are driven by substantive questions take on a double identity (being both a psychometrician and a psychologist) rather than identifying themselves as solely a psychometrician. For reasons cited earlier, people like Klaas Sijtsma, Susan Embretson, and Paul De Boeck are psychometricians who have a psychology-oriented approach.

#### The Consultant

Closely related to the psychology-oriented approach, but not entirely equivalent, is the consultant approach. The consultant aims to maintain a close relationship with psychologists and encourages collaborations, in which the psychologist comes up with a substantive research question, and the psychometrician offers methodological advice. The difference between the psychologist approach and the consultant approach is that the psychometricians of the first kind have an intrinsic interest in psychological theory and uses psychometrics as a way to build psychological theories, whereas the psychometrician with a consultant approach prefers to aid psychologists in solving methodological and statistical problems and leave the actual theory building to the psychologist. Peter Bentler and Bengt Muthén, who often collaborated with psychologists or other applied researchers and helped them solve complex methodological problems, might recognize themselves as taking up such a role in their research.

#### The Data Analyst

Third, we find that a number of presidents have more of a data analytic approach. These psychometricians view psychometrics as a toolbox that contains a set of models that are mostly of the latent variable type, which they consider applicable to a wide variety of data and disciplines. Though some of these models were perhaps originally designed for psychological measurement, in a data-analytic approach, these models are not necessarily used as substantive models and can be translated to several types of data for different types of purposes. The goals for the data analyst are usually not explaining the data or understanding the underlying mechanisms (which would be major motives for the psychology-oriented psychometrician) but rather to make predictions or summarize the data. Brian Junker, who, as quoted earlier, considers psychometric models to be translatable to all sorts of research problems. His view aligns with the data-analytic approach.

#### The Engineer

A fourth type we encountered is the engineer. Engineers are people who are interested in ‘making’ technologically advanced artifacts, which then find a clear application in society. Examples of such artifacts in psychometrics are innovative types of tests, like computer adaptive tests or simulation assessments, but also software programs. These applications then find their way to testing agencies, educational measurement, or the scientific community. Through these artifacts, the engineer may try to explain human behavior or solve challenging technical problems, but this takes place through a real-world application, rather than doing foundational or theoretical work only. People like Hua-Hua Chang, Wim van der Linden, and Robert Mislevy are co-builders of such applications and share an engineering-approach.

#### The Mathematician

Lastly, we distinguish the mathematician who gains most joy out of proving a mathematical theorem or solving a technical problem, without necessarily feeling the need to find an application or answering a substantive research question. The mathematician approach does therefore not require collaboration with psychologists or other applied researchers. For the mathematician, knowledge for the sake of knowledge (not for the sake of application) is sufficient. Moreover, the indisputable quality of mathematics—proving a theorem for once and for all—has an incredible appeal to some of the presidents. Jos ten Berge stresses that what he likes so much about psychometrics is ‘the absolute certainty with which you can decide about what is true or isn’t true. The mathematical part of it.’ This sentiment is also shared with Jan de Leeuw, who finds psychology too ‘debatable, or uncertain, or up in the air,’ and who appreciates the beauty of mathematics.

#### Two Dimensions of Psychometric Research

Naturally, a psychometrician does not necessarily fall under only one of the categories above: A combination of approaches is equally plausible. For example, someone who is a designer of technologically advanced tests—whom we might characterize as having an engineering approach—may also be interested in learning mechanisms in school children and thus have a substantive or psychological interest as well. For this reason, we summarize these categories in two dimensions, one ranging from ‘psychology’ to ‘statistics,’ the other ranging from ‘theoretical’ to ‘applied.’ Our respondents differ from each other in whether their research is driven by psychological questions or technical statistical issues, and at the same time, they differ in how strongly they concern themselves with applied or theoretical topics. Someone with a mathematical approach is more on the theoretical and statistical side of both dimensions, whereas the psychometrician with a strong interest in psychology can be located in the psychology/theoretical corner (or more on the applied side, if this psychometrician has a strong focus on doing applied research). These dimensions thus describe core aspects of the multifaceted identity of psychometric research.

### The Future of Psychometrics

The interviews provided an excellent opportunity to invite the presidents to take a look into the future of psychometrics and ponder on possible directions psychometrics might take. Some presidents think psychometrics will continue to remain relevant. Jos ten Berge stresses that since psychologists do not have the technical training that psychometricians have, there will always be a need for psychometricians. According to David Thissen: ‘[...] testing will continue to develop and continue to be a thing that is done for placement in education, in jobs. [...] I think testing still has some decades, if not centuries in it.’ Testing thus remains an important application of psychometrics. Analyzing test data well and making the right decisions based on test scores are still crucial in today’s society and will most likely continue to remain crucial in the upcoming decades. Moreover, testing now transcends traditional paper–pencil formats, and new types of tests are continuously being developed. The expertise of the psychometrician is therefore crucial and relevant and will remain so in the future.

However, the future relevance of psychometrics does not seem guaranteed. A number of interviewees express a certain sense of uncertainty with regard to a fruitful future of psychometrics. Though the interviewees disagree on what they believe the future holds, several presidents agree that a prosperous future for psychometrics is not a given. Psychometricians will have to put in the effort to make themselves relevant.

Some presidents point out that psychometrics has a serious PR problem and has to work hard to be heard, whether it is by psychologists or by other possible collaborators, and many see challenges in selling psychometric research to relevant parties. In fact, Wim van der Linden considers the inability of psychometrics to market itself as psychometrics’ biggest pitfall. He blames this inability on the slow development in psychometrics of making good user-friendly software, which would have paved the way for selling psychometric models at an earlier stage. Robert Mislevy states that ‘it is easier to get people to recognize the value and the use of psychometric techniques if you do not call them psychometric techniques until you have worked with them for a couple of months at least!’. Even though the presidents think it is crucial that psychometric knowledge is not lost to the test of time, psychometrics will have to make up a plan to remain influential. Mislevy continues: ‘there are very rapid advances today in technology, in psychology, in learning analytics, and the biggest challenge of psychometrics is not getting left in the dust.’

When asked about what the future holds for psychometrics, some respondents refer to the big data era, and how psychometrics could contribute to such new developments. Some say that the big data era provides an opportunity for psychometrics, and that again, we should not miss the boat. Ulf Böckenholt is full of optimism: ‘We live in the age of big data, the age of self-quantification. I carry a Fitbit. It is the dream of the psychometrician!’. And, according to Paul Holland, ‘The future of psychometrics is about the open-mindedness of all the different varieties of the ways that people collect data and try to draw conclusions and to make sense of it.’ It is the age of big data, and human response data are anything but extinct. In fact, more and more different types of data, in need of thorough analysis, are coming our way. And, according to Hua-Hua Chang, psychometricians have relevant knowledge that other researchers do not:

Everyone is talking about big data, but what is big data? How is the data collected? I think our psychometricians should do a good job of making sure data is collected reliably. How was the data collection designed? Does it have high validity? [...] That will make psychometricians even more important.

Thus, big data need to be analyzed appropriately, and psychometricians have the tools to get involved, also when the nature of these data is significantly different from traditional testing data.

But even though the big data movement seems more than promising, Jacqueline Meulman warns for the hype. According to Meulman, both psychometricians and statisticians should be critical of this development. Instead, psychometricians should claim back their own field:

They should say, ‘psychometrics is our area, and testing is from our origins, and we should claim it back.’ I am amazed sometimes by things I see on the Internet, that major agencies that do testing have no clue what psychometrics is all about.

Meulman stresses that it is by no means her intention to ignore developments that are going on in data science, but that it is essential to be on guard with these modern trends, and also to remain influential where psychometrics has always been needed the most: the testing industry. Ivo Molenaar also warns for the rise of big data: ‘I think that they [the psychometricians] have more computational possibilities now and have what they call big data [...]. I am getting old-fashioned, so I think maybe you should not collect that many data because it is only going to cause you problems.’ Molenaar refers here to the danger of overfitting and the lack of critical thinking in a mostly computer-driven process.

The future of psychometrics is thus regarded with careful optimism. Several presidents believe that psychometrics will remain relevant for psychology and the testing industry. But, where some presidents stress the importance of opening up to contemporary scientific ideas, others explicitly warn for these new developments. Both sides are afraid psychometrics might remain too isolated and out of touch with the scientific playground.

### Recommendations

Psychometrics might thus benefit from a change of course. But what change? It is challenging to extract a single recommendation from all twenty transcripts. What we can safely conclude is that contemporary psychometrics is essentially a pluralist research area, and it is this plurality that needs cherishing. This does not mean that we should just ‘let things be pluralist’ and each go our own ways, which is perhaps what is happening now. Instead, psychometrics needs to make explicit what a plurality of goals and approaches actually entails. What are the avenues that psychometrics aims to tread? What is psychometrics’ mission, and what are its priorities? Where and how does psychometrics want to contribute? We would recommend the Psychometric Society and other psychometric institutes to list their priorities and make a resulting mission statement public. Based on the interviews, these priorities could include: (1) building psychological theory, (2) improving educational measurement in terms of fairness or reliability, (3) constructing and distributing user-friendly software for the analysis of behavioral data, and (4) developing new methods for data analysis. Not only does such a list of priorities make it easier to communicate to external parties what it is that psychometrics does and values (something that worries many of our presidents), it can also offer guidance on relevant topics for sessions at meetings and the publication of articles. With this recommendation, we have no intention of preventing researchers from pursuing a path that is not listed as a priority. However, a more active policy may provide some clarity and guidance for a field that, if current trends continue, with time will only become more and more fragmented and diverse.

A second recommendation has to do with psychometrics’ relationship with its past and how its history also shapes contemporary psychometrics. Early psychometricians like Francis Galton, Lewis Terman, and James McKeen Cattell were often devoted to a specific social ideal—often associated with the highly controversial ideas of eugenics—and they expressed these ideals in their academic work. It is interesting to see that contemporary psychometricians do not often engage in public debate—even when educational measurement is again part of a heated discussion—and *Psychometrika *rarely publishes articles about such themes. Perhaps, psychometrics’ controversial history functions as a warning against a strong social involvement. Instead, contemporary psychometrics engages in highly technical work that, on the face of it, often seems to be detached from social reality. Psychometricians’ shyness for public expression does not help in improving their visibility, and importantly, it might lead to outcomes that are completely undesirable to the psychometrician (e.g., the possible decline of reliable measurement in schools or the rise of irresponsible data analysis). Whatever the reason for psychometrics’ current absence from public debate, we would recommend psychometricians to engage in matters that touch upon their expertise, not only as a way to increase their visibility, but more importantly, because they have expertise that matters.

## Conclusion and Discussion

First and foremost, the interviews testified to the fact that psychometrics is a multifaceted discipline, which creates tensions that are intrinsic to its organization: Psychometrics is structurally related to different fields, and our interviewees disagree on which of these affiliations should be leading. Clearly, modern-day psychometrics has evolved into a much more diverse, but also more fragmented field than it used to be in the early twentieth century when psychology was psychometrics’ main focus area. Though most psychometricians agree psychometrics may have a successful future, they also express worries about psychometrics not being able to reach out to other relevant areas.

The diversity of the field, both in current practice and perspectives on future directions, raises the question of how this diversity originates. One explanation for the diversity in psychometric research is that the Psychometric Society itself does not conduct a clear policy of where psychometrics should be heading. More than anything else, it is expertise and intellectual contributions that help decide whether someone becomes president of the Psychometric Society. Having a particular vision about the future of the field is not a requirement for the presidency. And even if a president is determined to adopt a specific policy in order to promote a particular approach of psychometric research, one year of presidency is often too short a period to leave a lasting impression on the psychometric research climate. The Psychometric Society, therefore, does not have one clear direction apart from promoting psychometric research in general and thus leaves plenty of room for a variety of approaches.

The diversity and fragmentation of psychometrics are not intrinsically problematic: The field is now reaching beyond its traditional boundaries, resulting in a wide variety of psychometric research projects all over the world, and that can certainly be considered a positive development for the field. But while the research topics and the psychometricians themselves have become more diverse and have only increased in number over time, psychometrics is clearly having difficulty with connecting to both its home base, psychology, and other relevant fields. If psychologists, statisticians, or other applied researchers do not seek out psychometric expertise when in need, psychometrics is in danger of becoming a field that is only practiced within its ivory tower, isolated from other research fields or social applications. For psychometricians with a theoretical or mathematical approach, this may not seem very problematic, but most psychometricians wish to contribute to some area of application, and for them, a further detachment between psychometrics and other scientific disciplines is not a desirable prospect. In this regard, psychometricians will have to find ways to improve communication with researchers from other areas, and perhaps the Psychometric Society and other organizations can play a more explicit role here (for instance, by making psychometrics journals more accessible to applied researchers or inviting a diverse group of speakers to speak at conferences). As we discussed in the section on recommendations, we would encourage the Psychometric Society to embrace psychometrics’ plurality and make explicit which goals psychometrics strives for, as to provide guidance in times of fragmentation. This is, of course, easier said than done, but for the sake of psychometrics’ future, it might be of vital importance.

Though this study does not have philosophical aspirations, the interviews and our analysis generate several questions about psychometrics that might be relevant for future studies into the history and philosophy of psychometrics. Earlier, we briefly alluded to the shift from classical test theory to modern test theory, which took place in the 1960s and 1970s, which was accompanied by the publication of Lord & Novick (1968). The first question that arises immediately is whether the transition from classical test theory and modern test theory can indeed be understood in Kuhnian terms. Is there indeed a drastic shift in the meaning of terminology from one paradigm to another, or do these paradigms coexist? Though Kuhnian or even Popperian readings of the history of psychometrics would indeed be valuable, our findings particularly invite a pragmatist reading of psychometric research. As we have shown in this paper, some psychometricians do not prioritize theory building, but rather the practical or predictive (pragmatic) value of a model or method. What it means for a psychometric model or method to be practical and how psychometrics is informed by a pragmatist philosophy are questions yet unanswered. A pragmatist reading of psychometrics would be particularly relevant since one of the inventors of pragmatism, William James (1907), is also one of the founding fathers of American psychology and psychometrics (Wijsen et al., 2019). His influence on the field, both in a historical and in a philosophical sense, has so far not received the attention it deserves.

Another topic worthy of further investigation is how psychometrics has become so diverse over time, and if it is representative of the diversification or specialization process in other fields. Can the patterns we found in psychometrics—an influx of researchers with a different educational background, less familiarity with traditional research topics, an increasing detachment between people with substantive interests and people who are more technology and statistics oriented, a more diverse playground of research topics—be generalized to other scientific domains? Is this a pattern that is almost typical for advanced discipline formation, or is psychometrics unique in that sense? All in all, though the transcripts themselves do not formulate answers to the questions stated above, they inspire further research in these areas, which we would certainly recommend.

### Some Limitations

An important limitation of this project is that we only invited presidents of the Psychometric Society as interviewees, who are well known in their field and have already received credit for their work, rather than psychometricians who are perhaps lesser-known and are still building a career for themselves. An oral history methodology would indeed recommend inviting people who have not already received plenty of attention for an interview since their voices are less frequently heard than the voices of people who stand at the forefront. Their ideas might be substantially different from those of our presidents. This is, of course, a valid point, and perhaps a future project could focus on reflections of psychometricians who are at the beginning of their careers. There is, however, a rationale behind choosing the presidents as our respondents. The presidents of the Psychometric Society have formed an integral part of twentieth and twenty-first-century psychometrics. Their ideas and views represent—though not exhaustively of course—historical and contemporary developments in psychometrics and are therefore intrinsically interesting and relevant.

A second limitation concerns the possibility that the interviewees did not want to be too explicit about certain topics, knowing the interview was to be videotaped and used for research. For the sake of avoiding unpleasantries, they may have avoided voicing unpopular opinions or pointing fingers. However, even if this was the case, we find plenty of explicit ideas and visions in the transcripts, giving us reason to believe that the presidents were more than willing to share their ideas about and memories of doing psychometric research.

Retrospectively, we find some questions and topics are left unaddressed or received too little attention in the interviews. Most notably, we find that the interviews lacked focus on the historical adverse effects of psychometrics on scientific research and society. Though briefly addressed in Sect. 2.2, we find that this highly relevant topic is deserving of a more thorough and critical investigation, and we recommend taking this up in further studies.

## Supplementary Information

Below is the link to the electronic supplementary material.

Supplementary material 1 (docx 17 KB)

## Footnotes

1 The modified transcriptions will become available in a separate publication (OMITTED FOR REVIEW, forthcoming); currently, transcriptions are available upon request.

## References

[CR1] Baer, M. A., Jewell, M., & Sigelman, L. (Eds.). (1991). *Political science in America: Oral histories of a discipline*. University Press of Kentucky.

[CR2] Wright C, Ville S (2017). The evolution of an intellectual community through the words of its founders: Recollections of Australia’s economic history field. Australian Economic History Review.

[CR3] Borsboom D (2006). The attack of the psychometricians. Psychometrika.

[CR4] Chitty, C. (2007). *Eugenics, race, and intelligence in education*. Continuum.

[CR5] Cronbach LJ (1951). Coefficient alpha and the internal structure of tests. Psychometrika.

[CR6] Cronbach, L. J., Gleser, G., Nanda, H., & Rajaratnam, N. (1972). *The dependability of behavioral measurements: Theory of generalizability for scores and profiles*. Wiley.

[CR7] Doel RE (2003). Oral history of American science: A forty-year review. History of Science.

[CR8] Dorans, N. J. (2011). Holland’s advice for the fourth generation of test theory: Blood tests can be contests. In N. J. Dorans & S. Sinharay (Eds.), *Looking back: Proceedings of a conference in honor of Paul W. Holland* (pp. 259–272). Springer.

[CR9] Groenen PJ, Van der Ark AJ (2006). Visions of 70 years of psychometrics: The past, present, and future. Statistica Neerlandica.

[CR10] Heiser W, Hubert L, Kiers H, Köhn F-F, Lewis C, Meulman J, McArdle J, Sijtsma K, Takane Y (2016). Commentaries on the ten most highly cited Psychometrika articles from 1936 to the present. Psychometrika.

[CR11] Herrnstein, R. J., & Murray, C. (1994). *The bell curve: Intelligence and class structure in American life*. Free Press.

[CR12] Inquirium, L. L. C. (2013). *Inqscribe: Digital media transcription software.* Retrieved August 30th, 2016, from http://www.inqscribe.com/.

[CR13] Jackson, J. P., & Weidman, N. M. (2004). *Race, racism, and science: Social impact and interaction*. ABC-CLIO.

[CR14] James, W. (1907). *Pragmatism: A new name for some old ways of thinking*. Harvard University Press.

[CR15] Jones, L. V., & Thissen, D. (2007). A history and overview of psychometrics. In C. R. Rao & S. Sinharay (Eds.), *Handbook of statistics: Psychometrics* (Vol. 26, pp. 1–27). Elsevier.

[CR16] Lord, F. M., & Novick, M. R. (1968). *Statistical theories of mental testing*. Addison-Wesley.

[CR17] Statistical theories of mental testing. Addison-Wesley.

[CR18] Sijtsma K (2006). Psychometrics in psychological research: Role model or partner in science?. Psychometrika.

[CR19] Smith R, Rennie D (2014). Evidence-based medicine–An oral history. Journal of the American Medical Association.

[CR20] Thompson, P. (2017). *The voice of the past: Oral history*. Oxford University Press.

[CR21] Thurstone LL (1934). The vectors of mind. Psychological Review.

[CR22] Traub RE (1997). Classical test theory in historical perspective. Educational Measurement.

[CR23] Van der Heijden PGM, Sijtsma K (1996). Fifty years of measurement and scaling and the Dutch social sciences. Statistica Neerlandica.

[CR24] Weiner C (1988). Oral history of science: A mushrooming cloud?. The Journal of American History.

[CR25] Wijsen LD, Borsboom D, Cabaco T, Heiser WJ (2019). An academic genealogy of Psychometric Society presidents. Psychometrika.

[CR26] Wijsen, L. D. (forthcoming). Twenty interviews with Psychometric Society presidents: What drives the psychometrician? Manuscript in preparation.

[CR27] Winston AS (1998). Science in the service of the far right: Henry E. Garrett, the IAAEE, and the Liberty Lobby. Journal of Social Issues.

[CR28] Abrams, L. (2010). *Oral History Theory*. Routledge.

[CR29] Young AG, Pearce S (2013). A beginner’s guide to factor analysis: Focusing on exploratory analysis. Tutorials in Quantitative Methods for Psychology.

[CR30] A beginner’s guide to factor analysis: Focusing on exploratory analysis. *Tutorials in 764 Quantitative Methods for Psychology*, 9, 79–94.

