# Supplementary material for: Perspectives on Psychometrics Interviews with 20 Past Psychometric Society Presidents
Source: Psychometrika. 2021 Mar 26;86(1):327–43. doi: 10.1007/s11336-021-09752-7 (PMC8035107; doi:10.1007/s11336-021-09752-7)
Supplement: Supplementary file 1 — Supplementary material 1 (docx 17 KB) [file 11336_2021_9752_MOESM1_ESM.docx]

| Name of Interviewee (Year of Presidency) | Year of Ph.D. Degree | Name of Advisor | University of Ph.D. Degree | Current Affiliation |
| --- | --- | --- | --- | --- |
| James O. Ramsay (1981) | 1966 | Harold O. Gulliksen | Princeton University | Professor Emeritus of Quantitative Modelling at McGill University |
| Peter M. Bentler (1982) | 1964 | Douglas N. Jackson | Stanford University | Distinguished Professor of Psychology and Statistics at UCLA |
| Lawrence Hubert (1983) | 1971 | Patrick C. Suppes | Stanford University | Professor Emeritus of Psychology, Statistics and Educational Psychology at the University of Illinois |
| Jan de Leeuw (1987) | 1969 | John P. van de Geer | Leiden University | Professor Emeritus of Statistics at UCLA |
| Bengt O. Muthén (1988) | 1977 | Karl G. Jöreskog | Uppsala University | Professor Emeritus of Education at UCLA |
| Paul W. Holland (1989) | 1966 | Patrick C. Suppes | Stanford University | Frederic M. Lord Chair in Measurement and Statistics in the Research and Development Division at the Educational Testing Service (retired) |
| Robert J. Mislevy (1993) | 1981 | R. Darrel Bock | University of Chicago | Professor Emeritus of Measurement, Statistics, and Education at the University of Maryland |
| Ivo W. Molenaar (1997) | 1970 | Jan Hemelrijk | University of Amsterdam | Professor Emeritus of Statistical Analysis and Measurement Theory for the Social Sciences at the University of Groningen (deceased) |
| Susan E. Embretson (1998) | 1973 | René V. Dawis | University of Minnesota | Professor of Quantitative Psychology |
| Wim J. van der Linden (1999) | 1980 | Gideon J. Mellenbergh | University of Amsterdam | Professor Emeritus of Measurement and Data Analysis at the University of Twente |
| David M. Thissen (2000) | 1976 | R. Darrell Bock | University of Chicago | Professor of Quantitative Psychology at University of North Carolina at Chapel Hill |
| William F. Stout (2001) | 1967 | Yuan Shih Chow | Purdue University | Professor Emeritus of Statistics at the University of Illinois at Urbana Champaign |
| Jacqueline Meulman (2002) | 1986 | John P. van de Geer | Leiden University | Professor of Applied Statistics at Leiden University |
| Willem J. Heiser (2003) | 1981 | John P. van de Geer | Leiden University | Professor Emeritus of Data Theory at Leiden University |
| Ulf Böckenholt (2005) | 1985 | R. Darrell Bock | University of Chicago | Professor of Marketing at the Kellogg School of Management at Northwestern University |
| Paul De Boeck (2007) | 1977 | Willem Claeys | Catholic University Leuven | Professor of Quantitative Psychology at Ohio State University |
| Brian W. Junker (2008) | 1988 | William F. Stout | University of Illinois | Professor of Statistics at Carnegie Mellon University |
| Jos M. F. ten Berge (2009) | 1977 | John P. van de Geer | University of Groningen | Emeritus Professor of Psychometrics at The University of Groningen |
| Klaas Sijtsma (2010) | 1988 | Ivo W. Molenaar | Groningen University | Professor of Psychometrics at Tilburg University |
| Hua-Hua Chang (2012) | 1992 | William F. Stout | University of Illinois | Professor of Educational Theory and Research Methodology at Purdue University |

Table 1 Details of interviews: Name, Year of Presidency, Year of Ph.D. Degree, Name of Advisor, University of Ph.D. Degree, and Current Affiliation.
